# Supplementary material for: A snapshot of selected neglected tropical disease research using the World Health Organization International Clinical Trials Registry Platform database, 1999–2023
Source: PLoS Negl Trop Dis. 2026 Jun 3;20(6):e0014338. doi: 10.1371/journal.pntd.0014338 (PMC13232815; doi:10.1371/journal.pntd.0014338)
Supplement: S1 Text — (DOCX) [file pntd.0014338.s002.docx]

In the first stage of the ICTRP search, the search terms were: “Buruli ulcer” OR “Chagas disease” OR dengue OR chikungunya OR dracunculiasis OR “Guinea-worm disease” OR echinococcosis OR “human African trypanosomiasis” OR “sleeping sickness” OR leishmaniasis or “kala-azar” OR leprosy OR “Hansen’s disease” OR mycetoma OR chromoblastomycosis OR mycosis OR mycoses OR onchocerciasis OR “river blindness” OR podoconiosis OR rabies OR scabies OR ectoparasitoses OR ectoparasite OR schistosomiasis OR “soil transmitted helminth” OR geohelminth OR “intestinal helminth” OR “intestinal nematode” OR “trichuris trichiura” OR “Ancylostoma duodenale” OR “Necator americanus” OR “Ascaris lumbricoides” OR roundworm OR whipworm OR hookworm OR strongyloides OR “snakebite envenoming” OR Taeniasis OR cysticercosis OR trachoma OR yaws OR Clonorchis OR Opisthorchiasis OR Paragonimiasis OR Fasciolasis OR “Lymphatic filariasis” OR ectoparasite.

An additional ICTRP search was conducted to include terms for deep mycoses: “sporotrichosis” OR “paracoccidiodomycosis”

Records were then subset in the second stage to the four NTDs of interest using the following search terms: “Chagas Disease”, “Chagas' Disease”, “Chagas”, “American Trypanosomiasis”, “South American Trypanosomiasis”, “Trypanosoma cruzi”, “Trypanosoma cruzi Infection”, “Trypanosoma cruzi Infections”, “T. cruzi”, “Kala-azar”, “Black Fever”, “Visceral Leishmaniasis”, “Leishmania”, “Schistosomiasis”, “Bilharziases”, “Bilharziasis”, “Katayama Fever”, “Schistoma Infection”, “Schistoma Infections”, “Schistosomiases”, “Schistosoma mansoni”, “Schistosoma japonicum”, “Schistosoma mekongi”, “Schistosoma guineensis”, “Schistosoma intercalatum”, “Schistosoma haematobium”, “Soil-transmitted helminthiases”, “Soil-transmitted helminth”, “Soil transmitted helminth”, “Geohelminth”, “Ascaris lumbricoides”, “Trichuris trichiura”, “Necator americanus”, “Ancylostoma duodenale”.
