# Supplementary material for: A snapshot of selected neglected tropical disease research using the World Health Organization International Clinical Trials Registry Platform database, 1999–2023
Source: PLoS Negl Trop Dis. 2026 Jun 3;20(6):e0014338. doi: 10.1371/journal.pntd.0014338 (PMC13232815; doi:10.1371/journal.pntd.0014338)
Supplement: S1 Table — Note that column percentages may add to 99.9% or 100.1% due to rounding to one decimal point. (DOCX) [file pntd.0014338.s003.docx]

|  | **Chagas Disease** | **Schistosomiasis** | **Soil-transmitted helminthiasis** | **Visceral leishmaniasis** |
| --- | --- | --- | --- | --- |
| **Number of unique studies** | 62 | 79 | 40 | 62 |
| **Study phase** |  |  |  |  |
| Phase I | 5  (8.1%) | 7  (8.9%) | 0 | 9  (14.5%) |
| Phase I/II | 2  (3.2%) | 1  (1.3%) | 1  (2.5%) | 1  (1.6%) |
| Phase II | 14  (22.6%) | 13  (16.5%) | 3  (7.5%) | 15  (24.2%) |
| Phase II/III | 2  (3.2%) | 2  (2.5%) | 0 | 2  (3.2%) |
| Phase III | 8  (12.9%) | 11  (13.9%) | 3  (7.5%) | 13  (21.0%) |
| Phase IV | 11  (17.7%) | 3  (3.8%) | 5  (12.5%) | 8  (12.9%) |
| Unclear | 5  (8.1%) | 6  (7.6%) | 8  (20.0%) | 2  (3.2%) |
| Not applicable | 15  (24.2%) | 36  (45.6%) | 20  (50.0%) | 12  (19.4%) |
| **Randomisation status** |  |  |  |  |
| Randomised | 46  (74.2%) | 57  (72.2%) | 32  (80.0%) | 39  (62.9%) |
| Non-randomised | 5  (8.1%) | 9  (11.4%) | 3  (7.5%) | 10  (16.1%) |
| Unclear | 6  (9.7%) | 9  (11.4%) | 3  (7.5%) | 12  (19.4%) |
| Not applicable | 5  (8.1%) | 4  (5.1%) | 2  (5.0%%) | 1  (1.6%) |
| **Intervention model** |  |  |  |  |
| Parallel | 39  (62.9%) | 31  (39.2%) | 21  (52.5%) | 41  (66.1%) |
| Single-arm intervention study | 8  (12.9%) | 13  (16.5%) | 4  (10.0%) | 14  (22.6%) |
| Cross-over | 7  (11.3%) | 1  (1.3%) | 0 | 0 |
| Factorial | 1  (1.6%) | 4  (5.1%) | 0 | 0 |
| Sequential | 0 | 4  (5.1%) | 0 | 1  (1.6%) |
| Unclear | 7  (11.3%) | 26  (32.9%) | 15  (37.5%) | 6  (9.7%) |
| **Blinding** |  |  |  |  |
| Open label | 27  (43.5%) | 36  (45.6%) | 18  (45.0%) | 47  (75.8%) |
| Blinded | 24  (38.7%) | 16  (20.3%) | 10  (25.0%) | 8  (12.9%) |
| Unclear | 11  (17.7%) | 27  (34.2%) | 12  (30.0%) | 7  (11.3%) |
