# Supplementary material for: A snapshot of selected neglected tropical disease research using the World Health Organization International Clinical Trials Registry Platform database, 1999–2023
Source: PLoS Negl Trop Dis. 2026 Jun 3;20(6):e0014338. doi: 10.1371/journal.pntd.0014338 (PMC13232815; doi:10.1371/journal.pntd.0014338)
Supplement: S2 Table — (DOCX) [file pntd.0014338.s004.docx]

| **Chagas disease (*n* = 88)** | **Single country** | **Multiple countries** | **Missing** |
| --- | --- | --- | --- |
| Phase I | 5 | 0 | 0 |
| Phase I/II | 2 | 0 | 0 |
| Phase II | 12 | 2 | 0 |
| Phase II/III | 2 | 0 | 0 |
| Phase III | 4 | 4 | 0 |
| Phase IV | 9 | 2 | 0 |
| Unclear | 28 | 3 | 0 |
| Not applicable | 13 | 2 | 0 |
| **Schistosomiasis (*n* = 102)** | **Single country** | **Multiple countries** | **Missing** |
| Phase I | 6 | 1 | 0 |
| Phase I/II | 1 | 0 | 0 |
| Phase II | 11 | 2 | 0 |
| Phase II/III | 2 | 0 | 0 |
| Phase III | 10 | 1 | 0 |
| Phase IV | 3 | 0 | 0 |
| Unclear | 44 | 5 | 0 |
| Not applicable | 13 | 3 | 0 |
| **Soil-transmitted helminthiases (*n* = 48)** | **Single country** | **Multiple countries** | **Missing** |
| Phase I | 0 | 0 | 0 |
| Phase I/II | 1 | 0 | 0 |
| Phase II | 3 | 0 | 0 |
| Phase II/III | 0 | 0 | 0 |
| Phase III | 2 | 1 | 0 |
| Phase IV | 3 | 2 | 0 |
| Unclear | 19 | 4 | 0 |
| Not applicable | 12 | 1 | 0 |
| **Visceral leishmaniasis (*n* = 84)** | **Single country** | **Multiple countries** | **Missing** |
| Phase I | 8 | 1 | 0 |
| Phase I/II | 1 | 0 | 0 |
| Phase II | 10 | 4 | 1 |
| Phase II/III | 2 | 0 | 0 |
| Phase III | 11 | 2 | 0 |
| Phase IV | 9 | 0 | 0 |
| Unclear | 20 | 4 | 0 |
| Not applicable | 10 | 1 | 0 |
