# Supplementary figures and images for: A snapshot of selected neglected tropical disease research using the World Health Organization International Clinical Trials Registry Platform database, 1999–2023
Source: PLoS Negl Trop Dis. 2026 Jun 3;20(6):e0014338. doi: 10.1371/journal.pntd.0014338 (PMC13232815; doi:10.1371/journal.pntd.0014338)

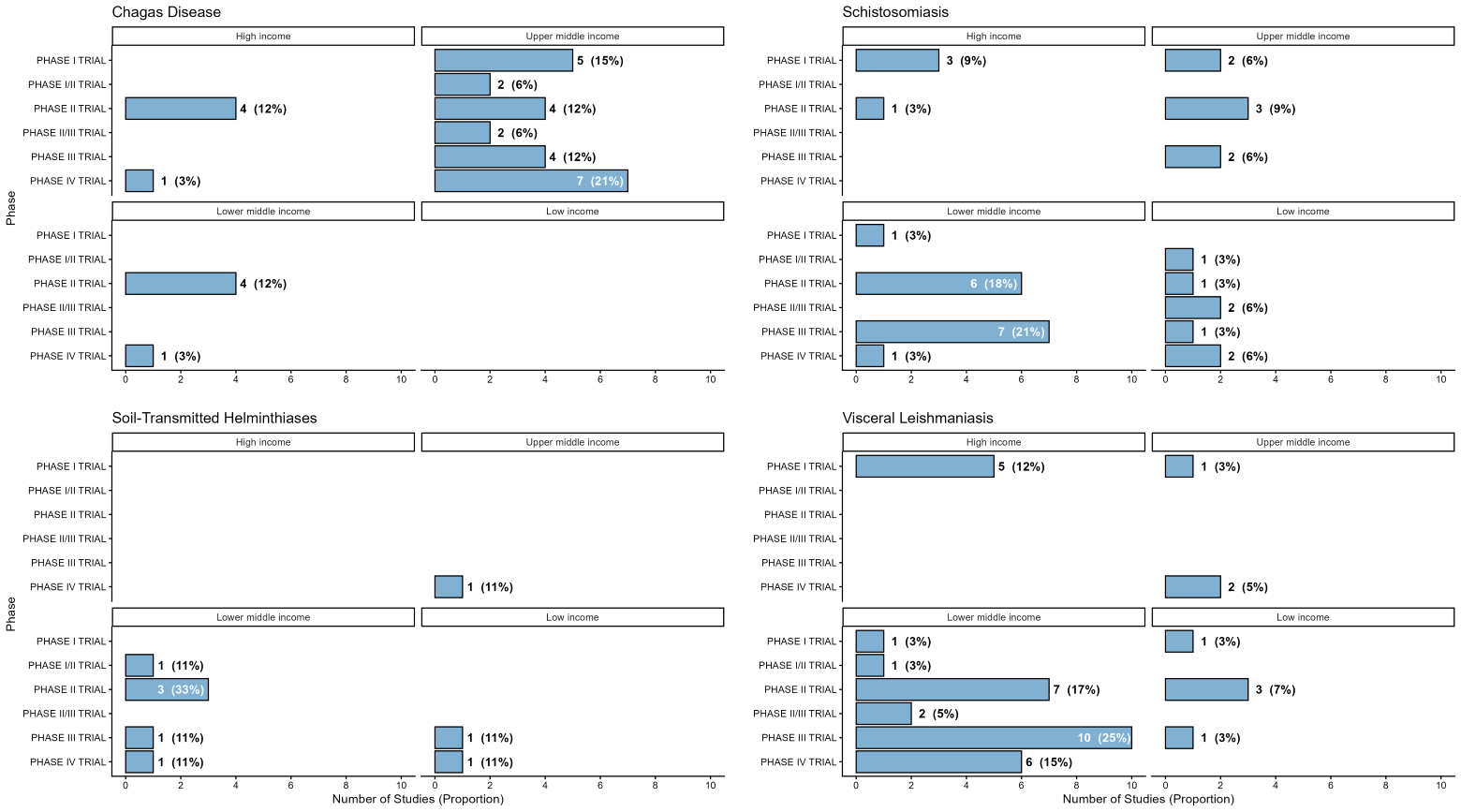

Supplement: S1 Fig — Length of bars represent the number of studies for each study phase, with annotated totals and proportion of total studies by disease. (TIF) [file pntd.0014338.s005.tif]

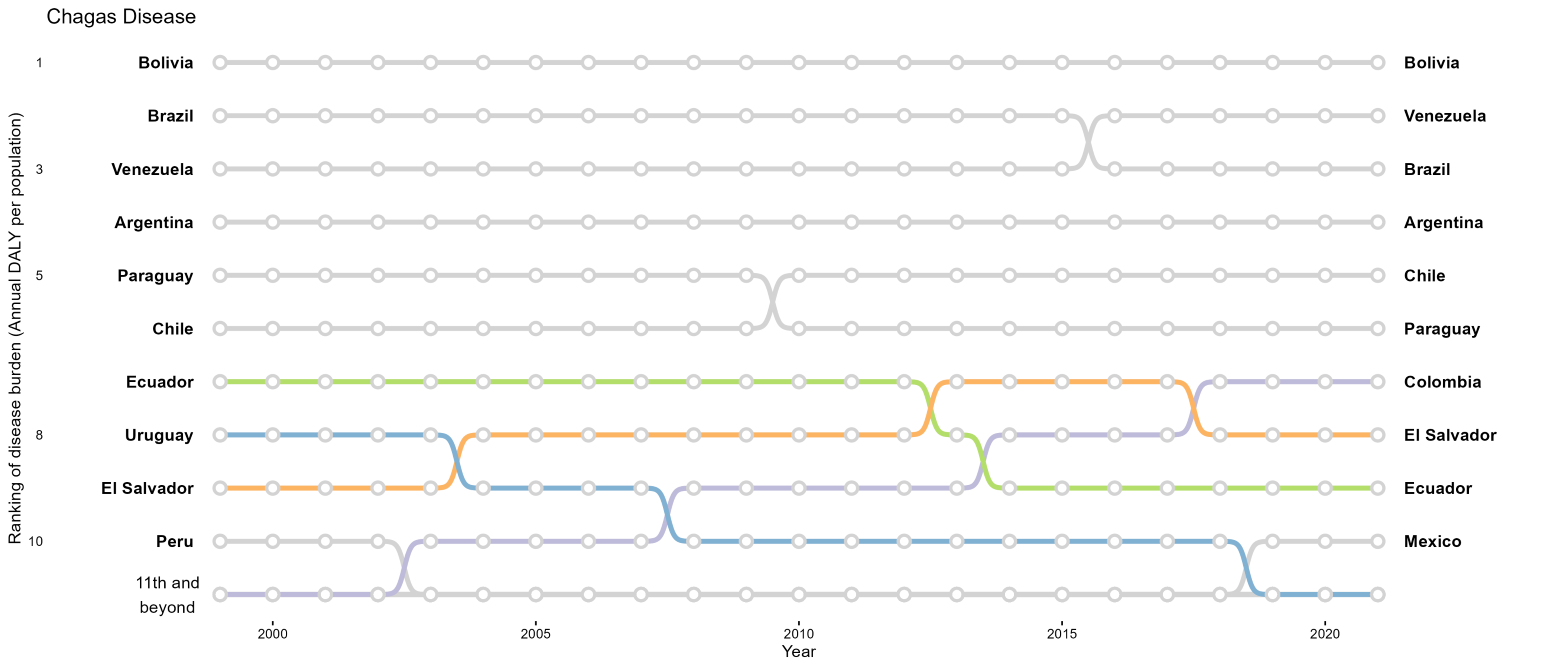

Supplement: S2 Fig — Disease burden is represented as the annual DALYs per million. Each dot is one year; countries are linked via edges/lines. Selected countries are highlighted in colours to show significant changes in ranking over the period 1999–2021 (2021 was the most recent burden data). (TIF) [file pntd.0014338.s006.tif]

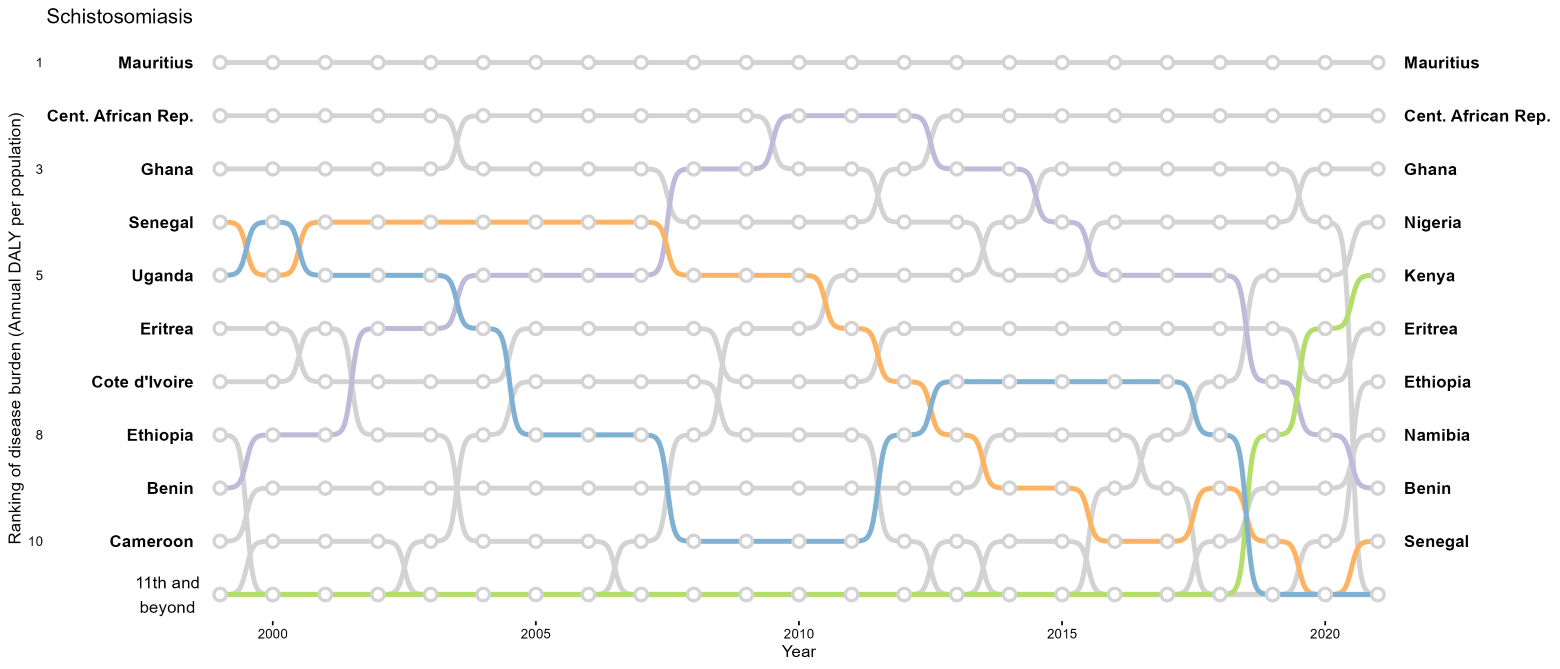

Supplement: S3 Fig — Disease burden is represented as the annual DALYs per million. Each dot is one year; countries are linked via edges/lines. Selected countries are highlighted in colours to show significant changes in ranking over the period 1999–2021 (2021 was the most recent burden data). (TIF) [file pntd.0014338.s007.tif]

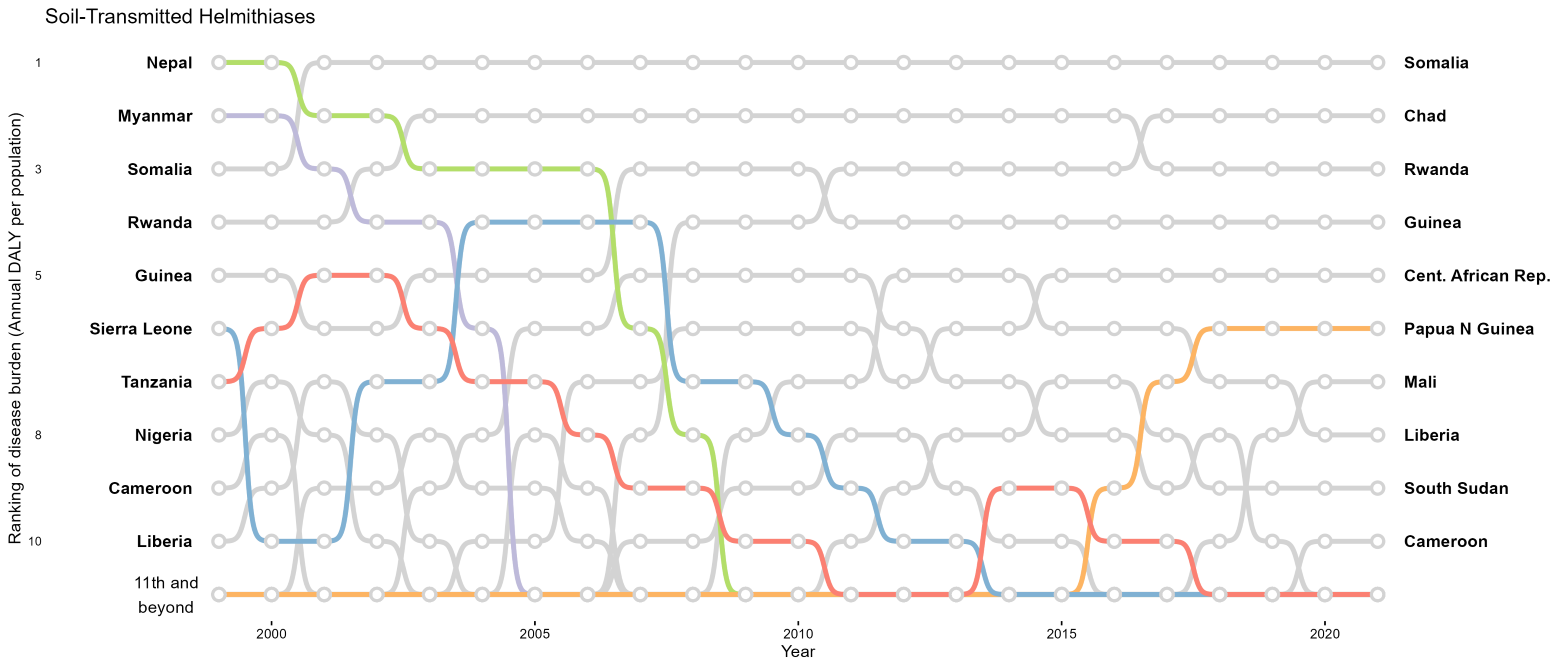

Supplement: S4 Fig — Disease burden is represented as the annual DALYs per million. Each dot is one year; countries are linked via edges/lines. Selected countries are highlighted in colours to show significant changes in ranking over the period 1999–2021 (2021 was the most recent burden data). (TIF) [file pntd.0014338.s008.tif]

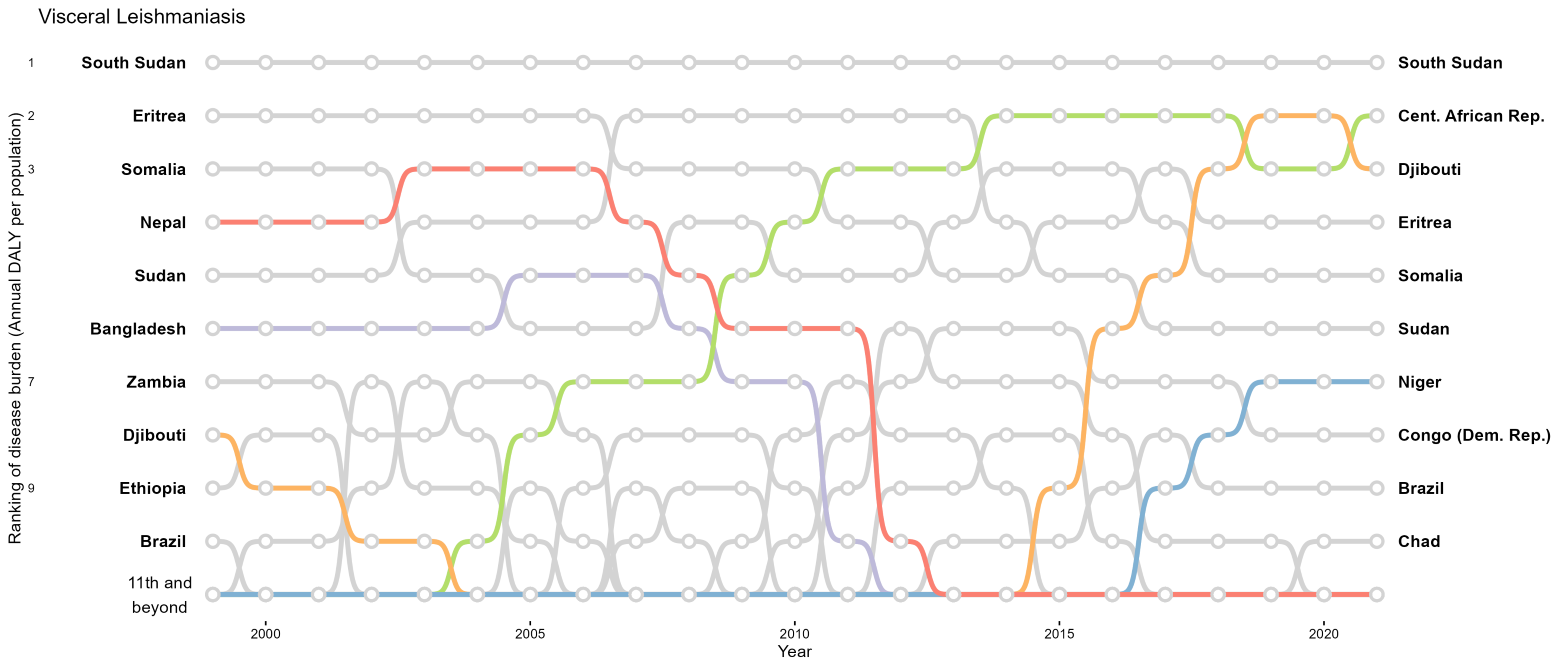

Supplement: S5 Fig — Disease burden is represented as the annual DALYs per million. Each dot is one year; countries are linked via edges/lines. Selected countries are highlighted in colours to show significant changes in ranking over the period 1999–2021 (2021 was the most recent burden data). (TIF) [file pntd.0014338.s009.tif]
