# Supplementary material for: A snapshot of selected neglected tropical disease research using the World Health Organization International Clinical Trials Registry Platform database, 1999–2023
Source: PLoS Negl Trop Dis. 2026 Jun 3;20(6):e0014338. doi: 10.1371/journal.pntd.0014338 (PMC13232815; doi:10.1371/journal.pntd.0014338)
Supplement: Attachment — Submitted filename: ICTRP NTD Response to Reviewers.docx [file pntd.0014338.s011.docx]

**Manuscript: ‘A snapshot of selected neglected tropical disease research using the World Health Organization International Clinical Trials Registry Platform database, 1999–2023’**

Submission ID: PNTD-D-26-00289

Dear handling editor,

Thank you for the opportunity to revise and re-submit our manuscript. The peer-reviewer’s report was helpful, and we believe the manuscript is better for the suggested changes.

Please see below the comments raised by the reviewers and the author team’s point-by-point responses.

Thank you for your time,

Rhys Peploe, Statistician

Infectious Diseases Data Observatory (IDDO)

[Rhys.peploe@iddo.org](mailto:Rhys.peploe@iddo.org)

**# Reviewer 1**

**#R1: Introduction**
Line 68 – include a clause that lists at least a few of the source registries that are included

**Authors’ reply:** An additional sentence has been added to name a handful of source registries. The following addition has been made on the tracked version of the manuscript:

Lines 70-73: “Source registries which provide data to ICTRP include: ClinicalTrials.gov, European Union Clinical Trials Register (EU-CTR), Pan African Clinical Trial Registry (PACTR) and International Traditional Medicine Clinical Trial Registry, amongst others.”

**#R1: Lines 78-79 – why were these 4 NTDs selected for this review? Could the authors include a justification?**

**Authors’ reply:** A justification has been added in the manuscript. The four diseases were chosen because of IDDO’s domain knowledge is stronger for these NTDs compared to others. The following sentence is added on the tracked version of the manuscript:

Lines 84-87: “These four diseases were chosen as IDDO remains actively engaged with the research community across these diseases, maintains a systematic scoping review of trials landscape, has supported the development of a global research agenda, and currently maintains a data platform of individual level data.”

**#R1: Methods**. Line 83 – data extraction section – were eligibility criteria for included studies pre-specified?

**Authors’ reply:** The criteria were pre-specified; this has been explicitly mentioned in the revised manuscript. The ‘Data extraction & curation’ subsection has been revised:

Lines 91-96: “The search to identify relevant records was completed in two stages; the eligibility criteria were pre-specified and agreed through discussions between IDDO and WHO. In the first stage, the ICTRP database was searched on 15 September 2023 to identify records on NTD which were published on or after 1 January 1999. Exclusion of records occurred if the condition was not an NTD, a vector study or human infection model where outcome was not related to disease or burden and if the focus of the study was not an NTD outcome, burden or treatment of an NTD.”

**#R1:** Line 83 – data extraction section – Did the authors consider collecting data on whether or not results were reported? Or otherwise, study status in general? It could be useful to know what percentage of studies that should have reported results indeed have reported results. Or the authors could clarify that results data are not included in the WHO registry in the same way that they are in others, like [clinicatrials.gov](http://clinicatrials.gov/) in more recent years

**Authors’ reply:** Study results and recruitment status are included in ICTRP, extracted from source registries such as clinicaltrials.gov. This was not part of the list of variables that was considered for this review. We have briefly looked at it and found only 13 of the 315 (4.1%) studies in Chagas disease, schistosomiasis, soil-transmitted helminthiases and visceral leishmaniasis reported results within ICTRP. However, further verification regarding whether the trial results were eventually published or not is required and not considered in this review.

Recruitment status is more populated than study results (only one missing data) however we could not see any indication that this data would be insightful and instead focussed resources into other more fascinating variables. Importantly, this variable does not mention whether studies were withdrawn or not, which would have been important.

**#R1:** Line 100 – disease burden, economic status, region classification section – for each indicator, please classify what geographic unit is being referred to. For example, based on the results, it looks like these indicators were extracted for countries specifically and later grouped into regions

**Authors’ reply:** Additional clarification to specify that indicators were extracted at country level, and these are indicated on lines 111, 116 & 118 of the tracked changes version of the manuscript.

**#R1:** Line 124 – I didn’t follow what “population access” meant in this context – could the authors elaborate?

**Authors’ reply:** Additional context has been added into this section to introduce the context and reasoning for this metric to clarify the objective of analysing population access to studies.

Lines 135-138: “Increased access to clinical studies is positive for several reasons. Study validity is improved as the sample population is likely to be more representative and diverse, whilst also allowing more people to be a part of research which can influence treatments and guidelines. To investigate this on a country level, population access to studies was defined as the total targeted (planned) number of participants per 10,000 population of the country over the period covered in the review (1999–2023).”

**Results**
**#R1:** Lines 134-135 – did the authors mean that the 462 articles were not focused on an NTD of interest (meaning, one of the 4 selected for this study)? Or not focused on NTDs at all?

**Authors’ reply:** These studies were not NTDs at all. This has been clarified in the manuscript.

Lines 148-150: “Of these, 462 (20%) were excluded because the disease of interest was not an NTD at all, many of the excluded studies were Leukaemia studies, for instance.”

**#R1:** Figure 4 – could the authors include more bubbles on the legend to give a sense of the size being represented by the smaller bubbles? The text indicates that Spain had only 6 annual DALYs per million, but the legend only gives sizes for 500 and 1000

**Authors’ reply:** More bubbles have been added to Figures 4, 6 & 7.

**#R1:** Lines 222-225 – did the 12 countries that saw declines in DALYs overlap with the decline in studies (did the same countries see fewer studies that saw declines in DALYs)?

**Authors’ reply:** An additional sentence has been added (lines 241-243, lines 271-273, lines 303-305, lines 338-340) to all four diseases detailing how the number of studies differs in 2004-13 to 2014-23 amongst the countries which experienced the highest burden and a reduction DALYs.

**Discussion**
**#R1:** Line 336 – now known as the Gates Foundation (no longer Bill & Melinda Gates Foundation)

**Authors’ reply**: Thank you. This has been changed in the manuscript (Line 367).

**#R1:** Lines 374-377 – I didn’t quite follow this logic. It seems to me that conducting a similar evaluation now for similar NTDs would support the development of the R&D framework by identifying gaps, as the authors have stated in the objectives previously, rather than the other way around

**Authors’ reply:** Thank you. These sentences have been redrafted in the revised manuscript as indicated.

Lines 407-410: “WHO is, at time of writing, leading work to develop a comprehensive R&D blueprint for NTDs; such a blueprint would benefit from future similar evaluations across over NTDs regarding the current research agenda and highlight priority areas for R&D investment”
